# Sulfate assimilation regulates hydrogen sulfide production independent of lifespan and reactive oxygen species under methionine restriction condition in yeast

**DOI:** 10.18632/aging.102050

**Published:** 2019-06-29

**Authors:** Kyung-Mi Choi, Sorah Kim, Seahyun Kim, Hae Min Lee, Alaattin Kaya, Bok-Hwan Chun, Yong Kwon Lee, Tae-Sik Park, Cheol-Koo Lee, Seong-il Eyun, Byung Cheon Lee

**Affiliations:** 1 College of Life Sciences and Biotechnology, Korea University, Seoul 02841, Republic of Korea; 2 Division of Genetics, Department of Medicine, Brigham & Women's Hospital and Harvard Medical School, Boston, MA 02115, USA; 3 Department of Culinary Art and Food Service Management, Yuhan University, Bucheon 422-749, Republic of Korea; 4 Department of Life Science, Gachon University, Sungnam, Republic of Korea; 5 Department of Life Science, Chung-Ang University, Seoul, Republic of Korea

**Keywords:** high-throughput genetic screening, methionine restriction, hydrogen sulfide, sulfate assimilation, reactive oxygen species

## Abstract

Endogenously produced hydrogen sulfide was proposed to be an underlying mechanism of lifespan extension via methionine restriction. However, hydrogen sulfide regulation and its beneficial effects via methionine restriction remain elusive. Here, we identified the genes required to increase hydrogen sulfide production under methionine restriction condition using genome-wide high-throughput screening in yeast strains with single-gene deletions. Sulfate assimilation-related genes, such as *MET1*, *MET3*, *MET5*, and *MET10*, were found to be particularly crucial for hydrogen sulfide production. Interestingly, methionine restriction failed to increase hydrogen sulfide production in mutant strains; however, it successfully extended chronological lifespan and reduced reactive oxygen species levels. Altogether, our observations suggested that increased hydrogen sulfide production via methionine restriction is not the mechanism underlying extended yeast lifespan, even though increased hydrogen sulfide production occurred simultaneously with yeast lifespan extension under methionine restriction condition.

## Introduction

Caloric restriction (CR) has been successfully shown to extend lifespans in various laboratory models [1]. The latest collaborative study between the National Institute on Aging and University of Wisconsin Madison involving rhesus monkeys reported an improvement in survival via CR [2]. Moreover, recent clinical trial in humans examined the effect of CR with respect to two well-known aging theories (rate of living and oxidative damage), which showed promising results in promoting human health by 2-year-CR [3]. However, despite sufficient evidence demonstrating CR-mediated longevity, it is difficult to maintain reduced caloric intake during an entire human lifetime. Thus, alternative strategies that mimic the CR effect without reducing total energy intake have been investigated. One of these strategies is methionine restriction (MR), a regimen that only limits nutritional access to methionine.

MR-mediated lifespan extension has been reported in short-lived organisms, including *Saccharomyces cerevisiae* (budding yeast) [4–7]. Two types of aging models exist in budding yeast: the replicative lifespan model measures the number of daughter cells produced by mother cells; and the chronological lifespan (CLS) model measures the survival time of populations during the stationary phase and is widely accepted as a model for postmitotic cell aging in higher organisms [8]. A recent study [9] showed that methionine-auxotroph yeasts (exhibiting defective *de novo* methionine biosynthesis) have longer CLS than prototroph strains capable of synthesizing methionine. In the same study, higher level of external methionine was observed to decrease CLS, whereas MR was shown to increase it.

Although the lifespan-extending effect of MR is well established across different species, not much is known about the mechanism of action by which MR elicits longevity. Recently Hine and coworkers [10] suggested CR-induced increased hydrogen sulfide production as a molecular mediator for CR-mediated lifespan extension. Hydrogen sulfide is produced via the transsulfuration pathway (TSP) enzymes, cystathionine β-synthase (CBS) and cystathionine γ-lyase (CGL). These enzymes are evolutionarily conserved across eukaryotes including yeast i.e., *CYS3* and *CYS4* encode *CGL* and *CBS*, respectively [11,12]. In addition to the TSP, a yeast-specific sulfate assimilation pathway also catalyzes extracellular sulfates into intracellular hydrogen sulfide. Once CR is induced in yeast (achieved by reducing glucose level from 2% to 0.5% in culture media), endogenous hydrogen sulfide production is observed to increase [10]. Interestingly, yeast strains lacking the assimilation pathway genes (*met5Δ*, *met14Δ*, and *met16Δ*) in 2% glucose media demonstrate decreased hydrogen sulfide levels. However, increased hydrogen sulfide via CR is still maintained in these mutants, thus implying the minimal involvement of the sulfur assimilation pathway in inducing hydrogen sulfide under CR conditions [10]. Given that MR and CR enhance longevity via nutrient restriction, we wondered whether MR possibly employed hydrogen sulfide to extend lifespan.

The goal of this study was to identify the genes or pathways that control hydrogen sulfide production under MR condition and to determine the role of hydrogen sulfide in lifespan extension. We investigated hydrogen sulfide production in nearly 3,500 knockout strains under regular or MR conditions using unbiased genome-wide high-throughput genetic screening. In contrast to that in CR conditions, we determined sulfate assimilation genes to be critical in increasing hydrogen sulfide production under MR condition. Furthermore, hydrogen sulfide induction was not associated with lifespan extension and decreased reactive oxygen species (ROS) under the MR condition. Altogether, this study suggests that the cellular function of hydrogen sulfide varies based on nutritional status, which may point out the difference between MR and CR while determining mechanisms for longevity.

## RESULTS

### High-throughput genetic screening-based identification of genes required for hydrogen sulfide production under MR condition

MR induces lifespan extension and triggers an increase in endogenous hydrogen sulfide levels [13]. Hydrogen sulfide is particularly considered to be an essential mediator of MR benefits; however, its role in MR still remains unclear. To address this, we exploited an MR media that was previously shown to extend the lifespan of yeast [6]. This MR media was first used to examine the change in hydrogen sulfide production in the presence of different methionine concentrations using a lead acetate paper assay. As expected, hydrogen sulfide production was observed to be consistently higher in yeast cells grown using MR media when compared to those grown using regular media (Fig. S1).

**Figure 1:**
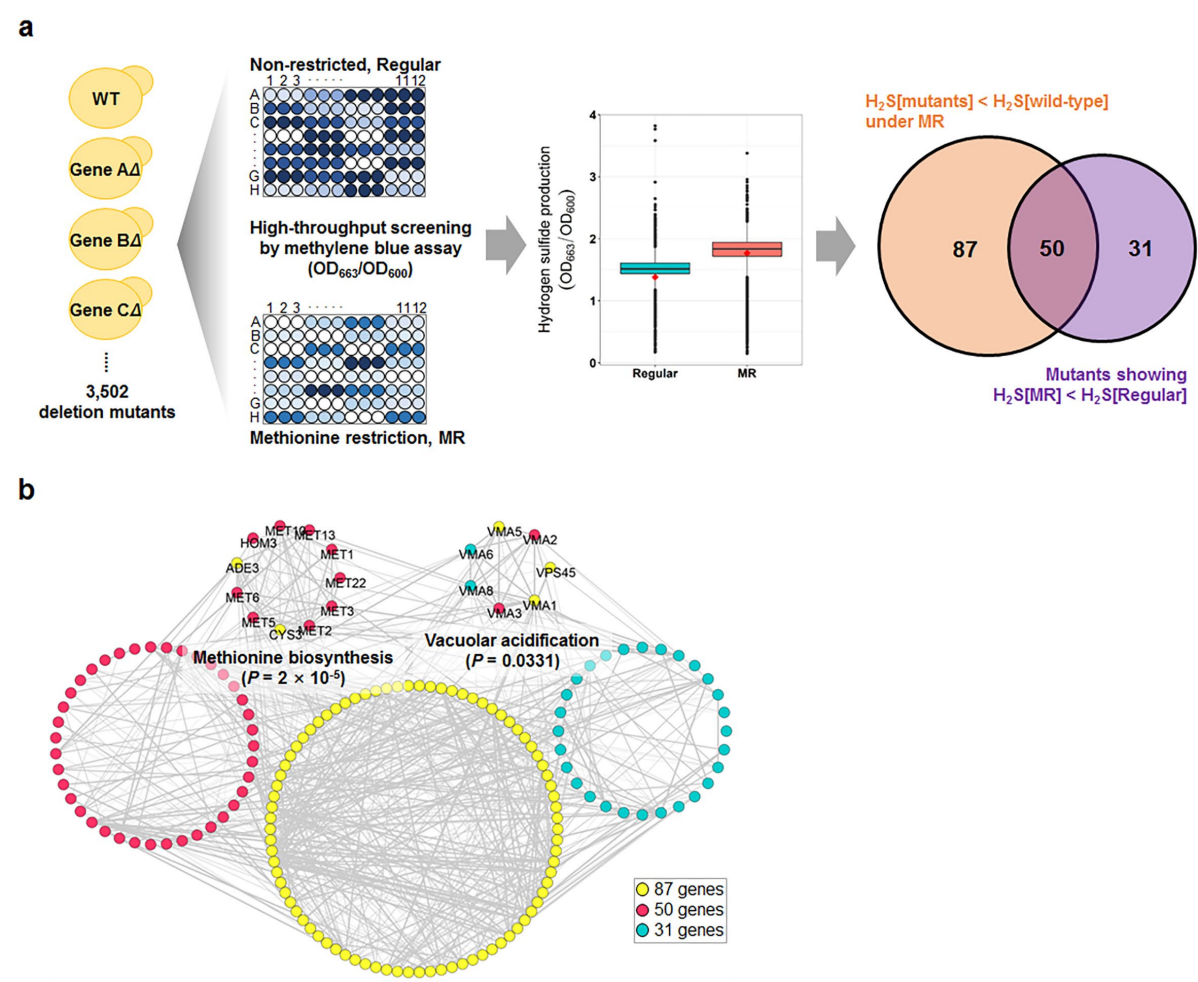
**Genome-wide screening for genes involved in hydrogen sulfide production under MR condition.** (**a**) Schematic screening strategy and hydrogen sulfide levels of total screening genes under regular and MR condition at 15 h after inoculation. The red diamond in the box plot indicates the value of the wild-type strain. The Venn diagram shows the number of mutants selected by 2 different statistical strategies. (**b**) Genetic network of the 168 selected genes from 2 analyses. The significantly enriched biological processes and involved genes are indicated.

Next, we conducted high-throughput hydrogen sulfide screening for the MR media with the yeast knockout collection (Fig. 1a). For this screening process, we used the methylene blue assay, in which the blue color changes to colorless upon hydrogen sulfide production. During this screening, two optical densities were measured simultaneously; one at OD_663_ to determine hydrogen sulfide production and another at OD_600_ to determine cell mass. These measurements were then used to calculate hydrogen sulfide production per cell. Compared to that in regular media, most of the tested strains, including wild-type, consistently showed low cell mass during the stationary phase (Fig. S2) and increased hydrogen sulfide production (Fig. 1a) in the MR media. Consequently, average hydrogen sulfide production per cell dramatically increased in the MR media. To identify the genes that regulated the increase in hydrogen sulfide production under the MR condition, two analytical strategies were applied to the screening data (Fig. 1a): 137 deletion mutant strains were selected to produce lesser hydrogen sulfide than the wild-type in MR media (Table S2; fold-change < -1.5; FDR-adjusted p-value < 0.05) and 81 deletion mutant stains were selected to exhibit no increase in hydrogen sulfide production under MR condition (Table S3; hydrogen sulfide production ratio between MR and regular media < 1; FDR-adjusted p-value < 0.05). Interestingly, 50 genes resided in the intersection between two independently performed comparisons (Fig. 1a). Next, we carried out Gene Ontology analysis for a total of 168 genes obtained from both analyses and found that genes involved in methionine biosynthesis (11 genes; p-value = 0.0000) and vacuolar acidification (7 genes; p-value = 0.0331) were significantly enriched (Fig. 1b). Therefore, we surmised that these two pathways (methionine biosynthesis and vacuolar acidification) possibly played a role in regulating hydrogen sulfide production under MR condition.

### Deletion of *MET1*, *MET3*, *MET5* or *MET10* reduces hydrogen sulfide production under MR condition

**Figure 2:**
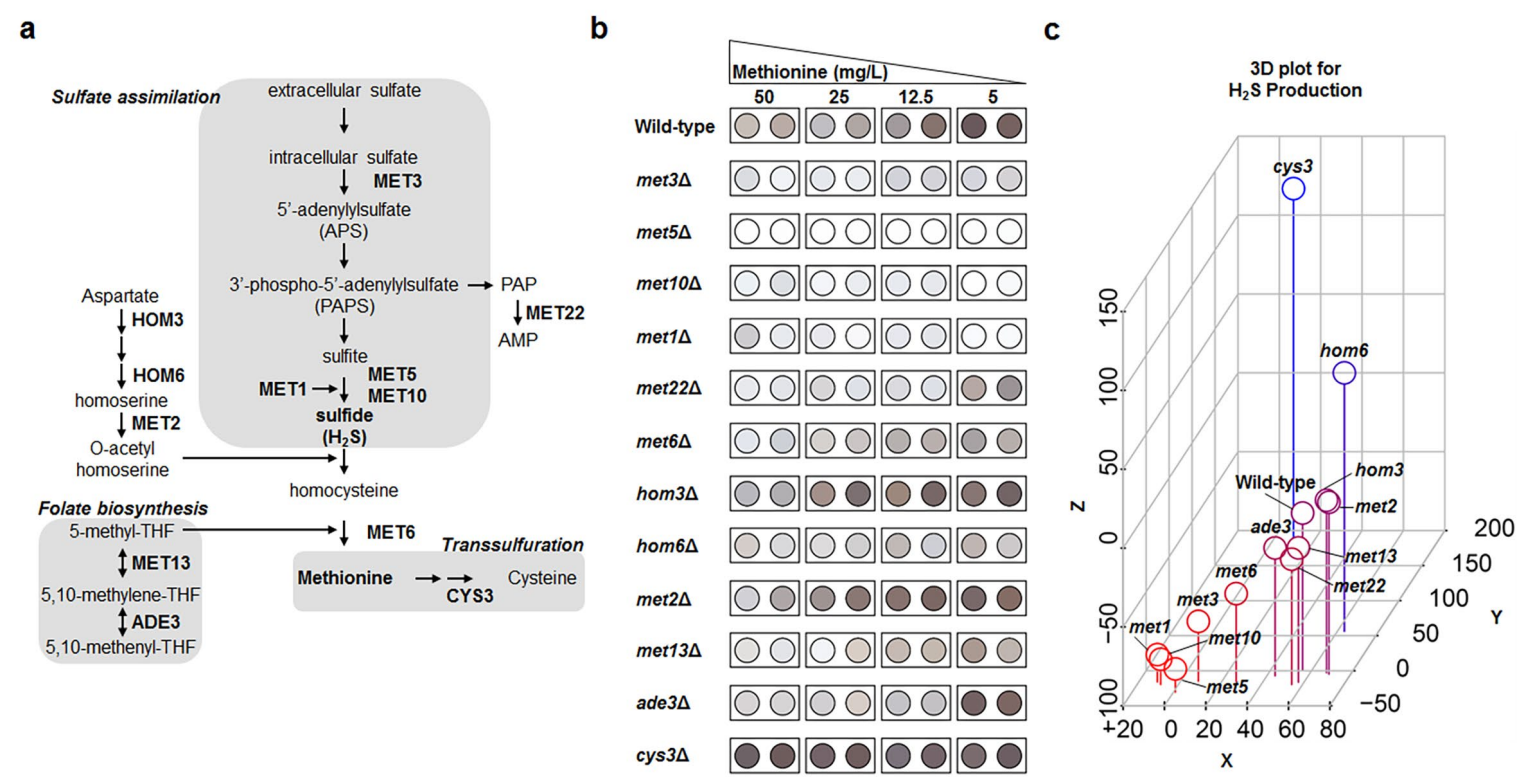
**Genes involved in sulfate assimilation are crucial for MR-mediated H_2_S production.** (**a**) Methionine metabolic pathway. A gene name is shown in the corresponding step. (**b**) Lead acetate assay to determine methionine metabolism-related genes under diverse methionine restricted conditions. (**c**) Three-dimensional plot demonstrating the effect of gene deletion on hydrogen sulfide production. Hydrogen sulfide levels under 50 mg/L (regular condition) and 5 mg/L (MR condition) of methionine were digitized based on spot darkness. For wild-type and deletion mutants, the x-axis indicates the difference between MR-condition and regular-condition hydrogen sulfide levels and the y- and z-axes indicate the difference between the deletion mutant and wild-type hydrogen sulfide levels under regular and MR conditions, respectively. Spot color shows low (red) to high (blue) z-axis values.

Based on the high-throughput methylene blue assay, methionine biosynthesis and vacuolar acidification were predicted to be involved in the regulation of hydrogen sulfide production under MR condition. Next, an alternative hydrogen sulfide detection assay was further performed to confirm these results owing to the pH dependency of methylene blue color change. Thus, we measured hydrogen sulfide production in deletion mutant strains associated with methionine metabolism (Fig. 2a) and vacuolar acidification under various methionine concentrations (50, 25, 12.5, and 5 mg/L) using lead acetate assay (Fig. 2b, S3, and S4). Methionine concentration showed a strong negative correlation with hydrogen sulfide production in wild-type yeast cells (Fig. 2b), which indicated reduced methionine uptake to be very effective in increasing hydrogen sulfide production. This phenomenon was also observed in most deletion mutant strains whose vacuolar acidification pathway genes were knocked out (Fig. S4). In contrast, all deletion mutant strains associated with sulfate assimilation, including *met1Δ*, *met3Δ*, *met5Δ*, and *met10Δ*, failed to increase hydrogen sulfide production to wild-type levels under MR conditions (Fig. 2b). These differences in hydrogen sulfide production were analyzed in following 3 ways and are shown in the 3D plot (Fig. 2c). For each strain, we subtracted the hydrogen sulfide amounts under regular condition from those under MR condition ([H_2_S]_MR_ – [H_2_S]_Reg_; x-axis). For regular condition, we subtracted the wild-type hydrogen sulfide amount from each deletion mutant hydrogen sulfide amount ([H_2_S]_mutant in regular_ – [H_2_S]_WT in regular_; y-axis). The values for z-axis ([H_2_S]_mutant in MR_ – [H_2_S]_WT in MR_) were calculated in the same way as those for the y-axis, except that they were obtained under MR condition. Interestingly, the four deletion mutant strains exhibiting defective sulfate assimilation were observed in the bottom left corner of the 3D plot, thus showing greatly reduced hydrogen sulfide production when compared to the wild-type under MR condition. Consequently, it also meant that the sulfate assimilation pathway was crucial for increasing hydrogen sulfide production under MR condition. Therefore, we surmised that the sulfate assimilation pathway may possibly have a regulatory role in upregulating hydrogen sulfide production when methionine uptake is reduced.

### Increased hydrogen sulfide production is not directly associated with lifespan extension of yeast under the MR condition

**Figure 3:**
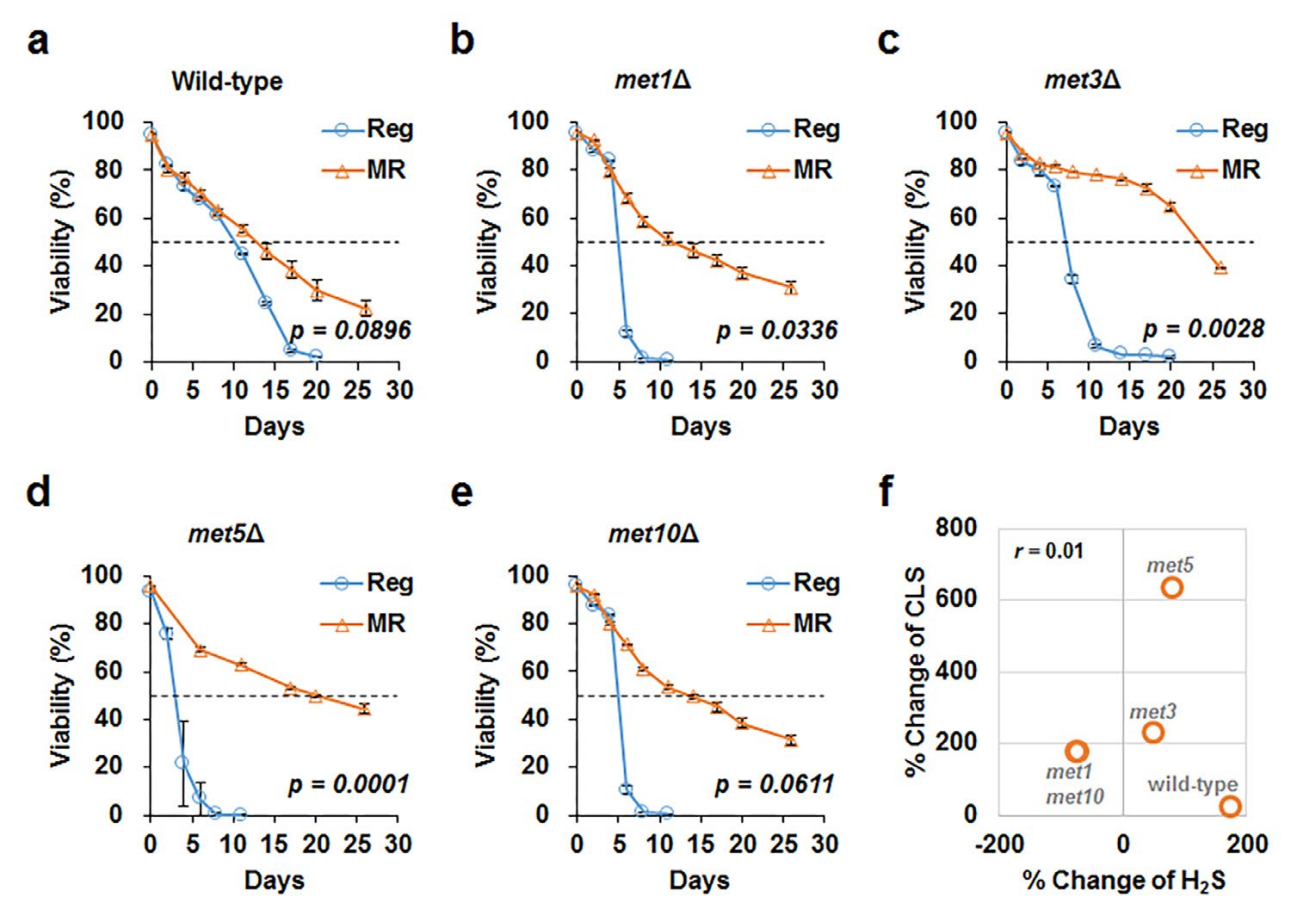
**MR successfully increases CLS despite defective sulfur assimilation.** CLS measured under regular (blue line) and MR (orange line) conditions in wild-type (**a**), *met1Δ* (**b**), *met3Δ* (**c**), *met5Δ* (**d**), and *met10Δ* (**e**) strains. The horizontal dotted line represents 50% viability. The graph indicates mean ± SEM. Statistical p-values between median CLS in regular and MR condition were calculated using two-tailed Student’s *t*-test. (**f**) For each strain, we plotted the change in hydrogen sulfide levels (x-axis) and change in CLS (y-axis) under MR conditions against regular condition. The Pearson’s correlation coefficient (r) was observed to be 0.01.

MR mimics many aspects of CR-mediated benefits, including lifespan extension and increased hydrogen sulfide production [10,13]. Increased hydrogen sulfide levels are particularly considered to be a mediator of lifespan extension under CR condition. In this regard, we assessed the effect of increased hydrogen sulfide production on lifespan under MR condition. Previously, DeLuna and his colleagues identified yeast aging genes from the stationary phase survival data of yeast single gene knockout mutants using an automated chronological lifespan assay [14]. Upon correlating our hydrogen sulfide production data against this genome-wide chronological lifespan data, no significant correlation between hydrogen sulfide production and lifespan was observed in the deletion mutant strains (correlation value was -0.009 and -0.151 under regular and MR conditions; Fig. S5). To demonstrate this experimentally, deletion mutants that exhibited defective sulfur assimilation and failed to increase hydrogen sulfide production in MR media were examined. Although these deletion mutants showed shorter lifespans than the wild-type in regular media, their lifespans were observed to extend at par with that of the wild-type under MR condition (Fig. 3 and Table S3). As a result, MR-mediated lifespan extension was concluded to be maintained independent of hydrogen sulfide production in all tested deletion mutants (Fig. 3f).

**Figure 4:**
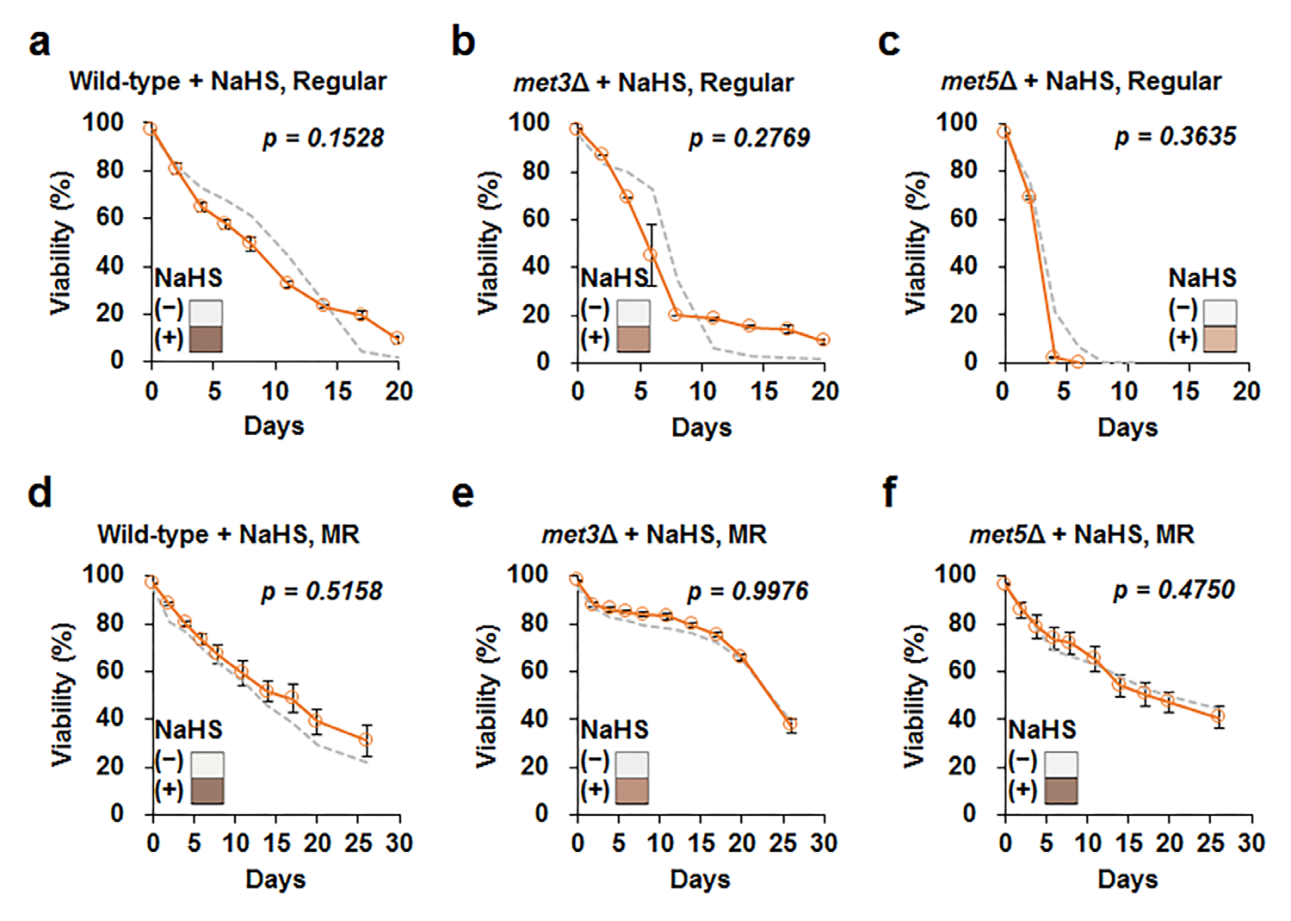
**Increase in exogenous H_2_S does not affect cellular lifespan.** Lifespan after addition of 50 μM sodium hydrosulfide (NaHS) at culture starting points (orange line) in wild-type, *met3Δ*, and *met5Δ* strains under regular (**a** to **c**) and MR (**d** to **f**) conditions. The graph indicates mean ± SEM. The gray dotted line indicates CLS without NaHS for each strain shown in Fig. 3. Statistical p-value between median CLS before and after NaHS addition was calculated using two-tailed Student’s *t*-test. The hydrogen sulfide levels measured using lead acetate paper is shown in the corner of the graph (original images are provided in Fig. S7).

To better understand the role of hydrogen sulfide in lifespan, we added a hydrogen sulfide donor, sodium hydrosulfide (NaHS), to wild-type cells grown in regular and MR media, i.e., the cells were treated thrice (6, 24, and 48 h after inoculation) with 5 µM NaHS. However, lifespan was not observed to extend by adding this small concentration of NaHS under both regular and MR conditions (Fig. S6). Next, we tested the effect of a much higher concentration of NaHS (50 μM) on wild-type, *met3Δ*, and *met5Δ* strains at the culture starting point (Fig. 4 and Fig. S7). Whereas NaHS markedly increased hydrogen sulfide production, lifespan remained unchanged after NaHS treatment in all tested strains under regular and MR conditions. Therefore, we concluded that increased extracellular hydrogen sulfide production was not directly related to lifespan extension under the MR condition.

**Figure 5:**
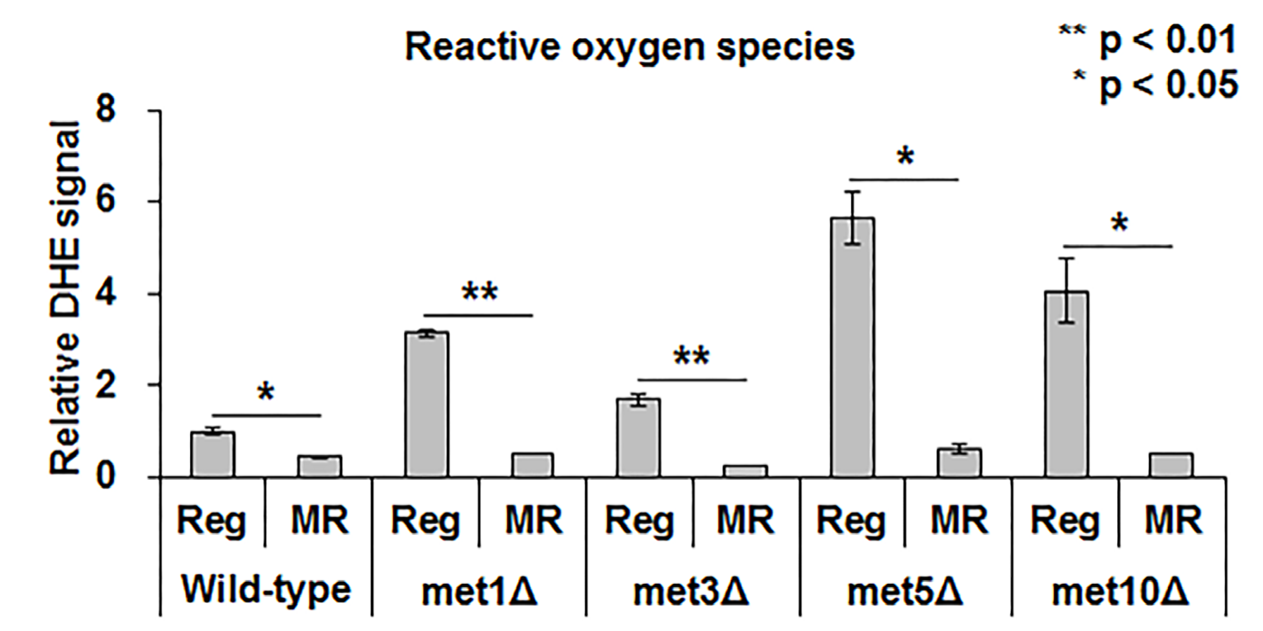
**MR significantly reduces ROS regardless of methionine assimilation defectiveness.** ROS levels in the wild-type strain under regular condition is considered as the standard. The relative level of the other strains is depicted. Experiments were run in triplicate. The bar graph indicates the mean ± SEM. The asterisks (*) indicate the p-value calculated using two-tailed Student’s *t*-test between regular and MR conditions in the same strain.

Recent studies suggested that hydrogen sulfide protected cells from oxidative stress by reducing ROS or increasing antioxidant production [15]. Hence, we examined the relation between ROS and MR-mediated lifespan extension and hydrogen sulfide production. Under regular condition, deletion mutants produced about 2 to 6-fold higher ROS levels than the wild-type (Fig. 5). However, no noticeable difference in the ROS levels was observed between mutants and the wild-type upon methionine restriction (Fig. 5). Thus, all strains, including the wild-type and deletion mutants, showed decreased ROS levels via MR. Although this suggested a relation between MR-mediated decrease in ROS levels and lifespan extension under MR condition, it did not necessarily associate the process with changes in hydrogen sulfide production via MR.

## DISCUSSION

In this study, we demonstrated that MR promotes hydrogen sulfide production similar to CR [10]. However, unlike CR, the induced hydrogen sulfide does not correlate with lifespan extension via MR, thus questioning the beneficial effects of MR on longevity via hydrogen sulfide production.

As a systematic approach to identifying genes involved in hydrogen sulfide production under MR condition, we screened nearly 3,500 knockouts in the BY4741 yeast genetic background. Both, a methylene blue assay for genetic screening (Fig. 1) and a sensitive lead-acetate assay (Fig. 2) allowed us to observe the positive role of the sulfate assimilation pathway in hydrogen sulfide production under MR condition. Sulfate assimilation-deficient strains showed a drastic reduction in hydrogen sulfide production when compared to the wild-type strain under both MR and regular conditions. However, the effect of deletion of the sulfate assimilation genes with respect to hydrogen sulfide production was much stronger under MR condition. Furthermore, a gradual increase in hydrogen sulfide levels along with an increase in the MR extent was abolished in deletion strains defective for sulfur assimilation (Fig. 2). Previously, *MET1* and *MET5* gene expression was reported to be induced upon methionine limitation [16]. Based on this report, we surmise that sulfur assimilation-related gene transcription may be induced and the pathway leading to *de novo* methionine biosynthesis may also be activated when methionine supplementation is reduced. This may be a cellular adaptation process to compensate for the low intracellular methionine levels. As a result, this may increase hydrogen sulfide levels, which is produced during the process of converting extracellular sulfate to homocysteine. In this context, cells lacking sulfate assimilation genes may fail to activate the sulfate assimilation pathway, thereby resulting in decreased hydrogen sulfide production under MR condition.

Although our initial screening data also showed the involvement of vacuolar acidification in regulating hydrogen sulfide production under MR condition (Fig. 1b), this process was not identified in the lead-acetate assay (Fig. S4), thus suggesting that pH variation caused by vacuolar acidification gene deletion may have influenced the methylene blue assay results. Intriguingly, a recent study suggested that autophagy-dependent vacuolar acidification was required for lifespan extension via MR [9]. Disruption of vacuolar acidification via *ATG5* deletion in the methionine-auxotrophic *met2Δ* strain (*met2Δatg5Δ*) abolished lifespan extension in the *met2Δ* strain when compared with that in the methionine-prototrophic MET^+^ strain. Furthermore, overexpression of vacuole ATPases, including *VMA1* and *VPH2* (which leads to vacuole acidification), increased CLS in the MET^+^ strain and did not extend the lifespan in *met2Δ*, thus suggesting the promotion of MR-mediated longevity via enhanced vacuolar acidity. However, no difference in hydrogen sulfide production under MR condition was observed between the wild-type and various deletion mutants devoid of vacuolar acidification (Fig. S4). Thus, our data again pointed out that MR likely extended yeast lifespan independent of hydrogen sulfide.

In animals, the breakdown of cysteine via *CBS* and *CSE* is crucial for hydrogen sulfide production; therefore, those two genes play a role in controlling endogenous hydrogen sulfide production. In yeast, *CYS3* and *CYS4* are orthologs of mammalian *CSE* and *CBS*, respectively. Interestingly, hydrogen sulfide production in *cys3Δ* (Fig. 2b) and *cys4Δ* was higher than that in the wild-type under both regular and MR condition [17]. Consequently, it suggests that the defective cysteine catabolism may be related to the upregulation of sulfate assimilation in order to compensate for hydrogen sulfide production. Importantly, sulfate assimilation via sulfite reductase is not conserved in animal, while cysteine catabolism via *CBS* and *CSE* acts as the main source of hydrogen sulfide [18]. Thus, we hypothesize that a different strategy might be adopted to regulate hydrogen sulfide production in yeast under MR condition and it might be the reason why increased hydrogen sulfide production by MR is not the mechanism underlying extended yeast lifespan.

Previously, hydrogen sulfide was suggested to alleviate oxidative stress levels by reducing ROS generation [19,20]. However, our data (Fig. 5) showed that ROS generation under MR condition remained consistently low regardless of hydrogen sulfide levels, thereby supporting the idea that yeast, unlike mammals, utilize a different mechanism to regulate hydrogen sulfide production under MR condition. Consistently, lifespan extension also does not correlate with the regulation of hydrogen sulfide production under MR condition (Fig. 3 and 4). In conclusion, yeast uses a different strategy, such as sulfate assimilation, to increase its hydrogen sulfide production against methionine deficiency, which is independent of longevity and ROS generation. Future work involving an unbiased systematic approach in other species should be carried out to further clarify the origins of the beneficial effects of MR.

## MATERIALS AND METHODS

### Yeast strains and culture media

*Saccharomyces cerevisiae* BY4741 (MATa *his3Δ*1 *leu2Δ*0 *met15Δ*0 *ura3Δ*0) and deletion strain collections (BY4741 background) were used in this study (EUROSCARF, Germany). Unless otherwise noted, yeast cells were grown in synthetic medium (Table S4) containing 50 mg/L or 5 mg/L of methionine for regular and MR conditions, respectively [21].

### High-throughput genetic screening for hydrogen sulfide production

Hydrogen sulfide screening was conducted using methylene blue assay as described by Winter G and Curtin C [22] with minor modifications. Briefly, cells were precultured to the stationary phase in 200 μL of YPD (1% yeast extracts, 2% peptone, and 2% glucose) prior to inoculation. Assays were performed in a microtiter plate at a total volume of 250 μL per well. Each well contained 247 μL of medium and 3% methylene blue reaction mix [23]. The experiments were carried out in triplicate.

Two optical densities were measured: OD_663_ for the hydrogen sulfide indicator and OD_600_ for the cell mass of the wild-type and 3,502 single gene deletion strains. The raw data for hydrogen sulfide production were calculated using the following formula:

Hydrogen sulfide production of strain A = OD_663_ of strain A / OD_600_ of strain A

### Selection of genes involved in hydrogen sulfide production under MR condition

To prevent bias from growth defective strains, we eliminated mutant strains exhibiting OD_600_ values less than one-third the wild-type OD_600_ value (Fig. S8). To identify single gene deletion mutants showing lower hydrogen sulfide production than the wild-type under MR condition, we compared the fold-change between mutant and wild-type hydrogen sulfide production and determined the p-value using Student’s *t*-test followed by false discovery rate (FDR) adjustment. When the adjusted p-value was less than 0.05 and the fold-change between mutant and wild-type was less than -1.5, genes (137 gene deletion strains; Table S1) were categorized to be significantly involved in hydrogen sulfide production under MR condition. We also compared hydrogen sulfide production in each mutant strain under MR and control conditions. When the ratio of hydrogen sulfide production between the MR and control conditions was less than 1.0 and FDR-adjusted p-value was less than 0.05, genes (81 gene deletion strains; Table S2) were categorized to be significantly involved in hydrogen sulfide increase via MR.

### Lead acetate assay

Commercially prepared lead acetate strip papers (Merck Millipore, Germany) or manually prepared lead acetate strip papers (Whatman filter paper soaked in 300 mM lead acetate solution and then dried) were used to detect the hydrogen sulfide emitted from the yeast batch culture. The paper was attached to the culture flask lids and incubated for 1 to 12 h at 30 °C until the paper darkened (due to lead sulfide formation). A color representation from the raw images (Fig. S3) was picked using Photoshop and is presented (Fig. 2b).

### Chronological lifespan assay

Lifespan assay was conducted using propidium iodide staining as described previously [24,25]. Three yeast colonies of each yeast strain were seeded into 10 mL of 2% glucose-containing rich media (YPD) and incubated overnight. The seed culture was re-inoculated into 20 mL of YPD and the yeast cells were grown until the end of the lifespan assay. As the indicated time-point on the viability graph, cells were harvested, washed with phosphate-buffered saline (PBS). And stained with 5 µg/mL of propidium for 20 min at 30 °C. Fluorescence was detected using FACS Verse (BD, USA).

### ROS measurement

Cells were harvested at day 6 and incubated in PBS solution containing 50 μM dihydroethidium (Sigma, USA) at 30°C for 20 minutes. Samples were washed thrice with PBS solution and resuspended in 1 mL of PBS. Fluorescence signal was detected in the FL3 channel using flow cytometry (FACSVerse).

## SUPPLEMENTARY MATERIAL

Supplementary Figures

Supplementary Tables
