# Supplementary material for: Sulfate assimilation regulates hydrogen sulfide production independent of lifespan and reactive oxygen species under methionine restriction condition in yeast
Source: Aging (Albany NY). 2019 Jun 29;11(12):4254–73. doi: 10.18632/aging.102050 (PMC6628990; doi:10.18632/aging.102050)
Supplement: Supplementary Figures [file aging-11-102050-s002.pdf]

SUPPLEMENTARY MATERIAL

Supplementary Figures

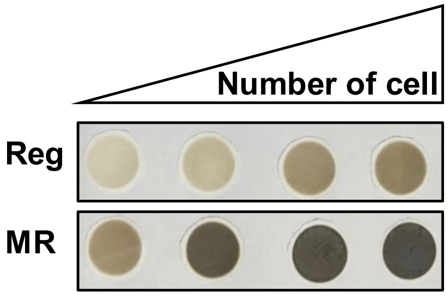

Figure S1. Hydrogen sulfide production by increasing the number of cells under regular (Reg) and MR condition. Hydrogen sulfide was detected by lead acetate method.

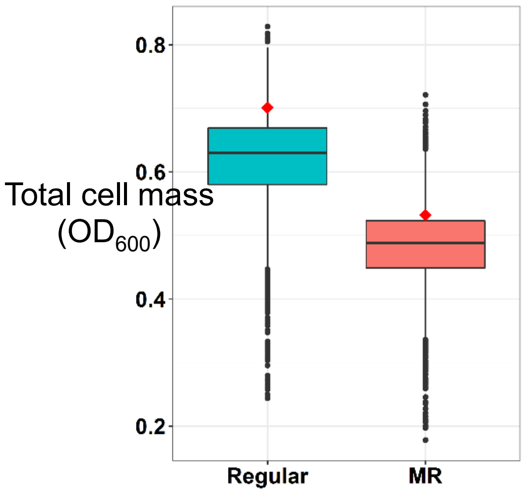

Figure S2. OD_{600} of total screening genes under regular and MR condition at 15 hours after inoculation. The red diamond indicates value for wild-type.

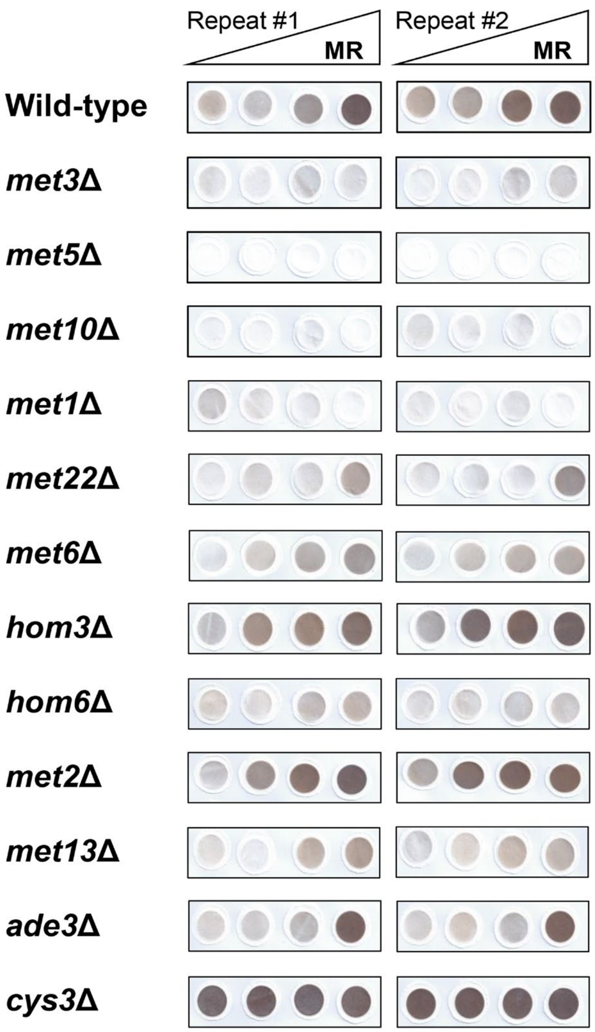

Figure S3. Original images for lead acetate assay in Fig.

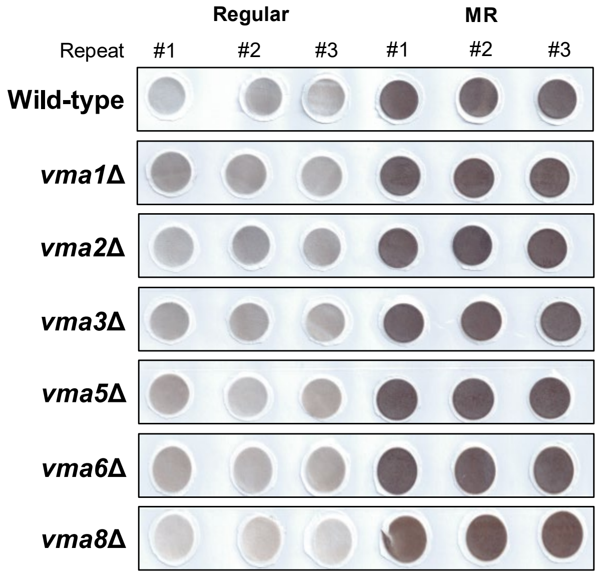

Figure S4. Lead acetate assay for mutants related with vacuolar acidification.

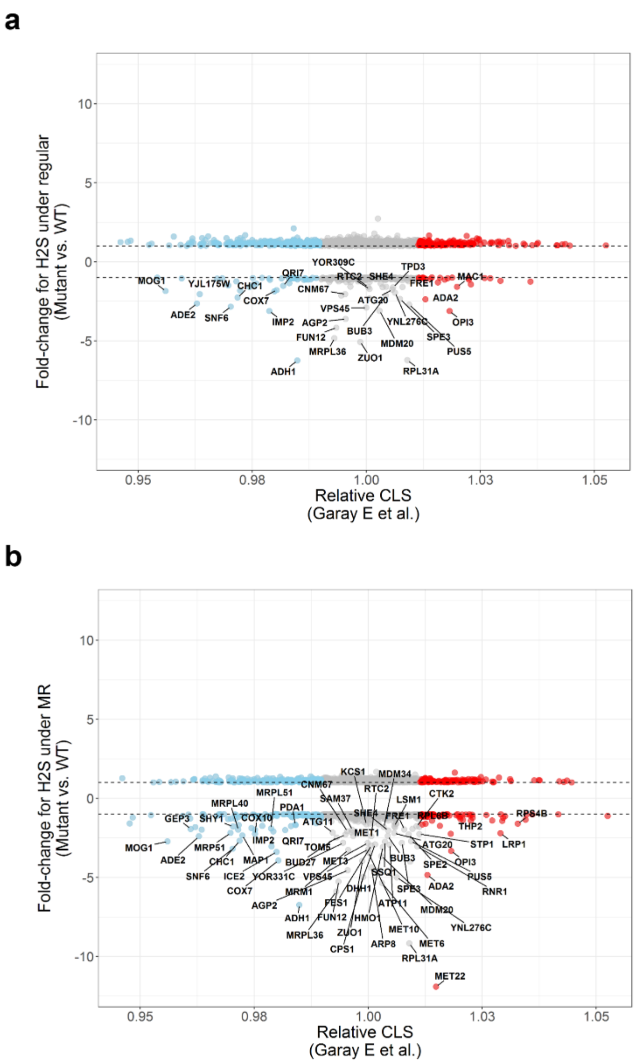

Figure S5. **Comparison between our hydrogen sulfide screening data and chronological lifespan (CLS) screening data by Garay E et al.** Compared to wild-type, change of hydrogen sulfide amount in deletion mutants under regular (a) and MR (b) is depicted. Black dotted line indicates a point where absolute value of fold-change (y axis) is "1". Black label indicates selected genes by comparison between wild-type and mutant under each condition in this study (fold-change < -1.5 and FDR-adj p < 0.05). According to relative CLS, strains with significantly decreased or increased lifespan are shown in blue or red, respectively.

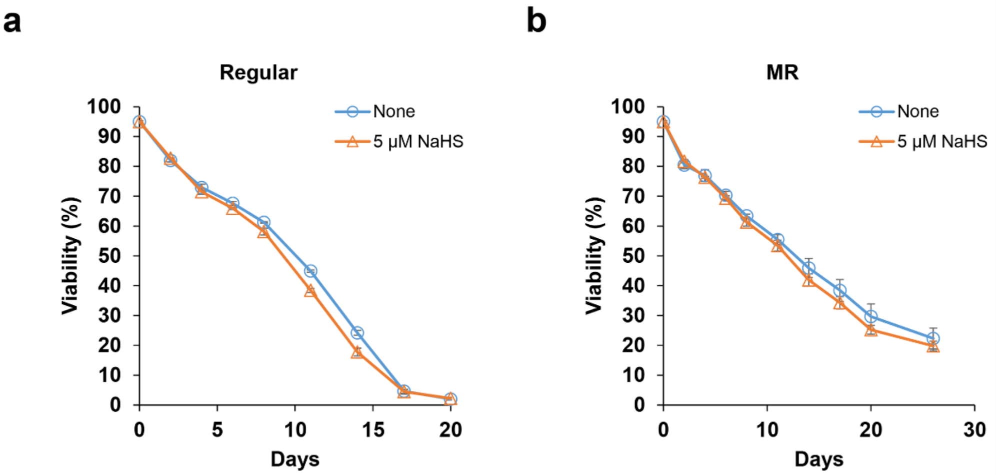

Figure S6. Lifespan under regular (**a**) and MR (**b**) before (blue line) and after (orange line) treating 5 µM NaHS. Graph indicates mean ± SEM.

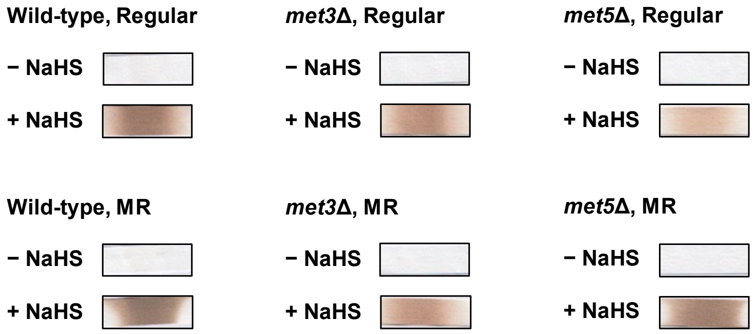

Figure S7. Original images for lead acetate assay in Fig. 4.

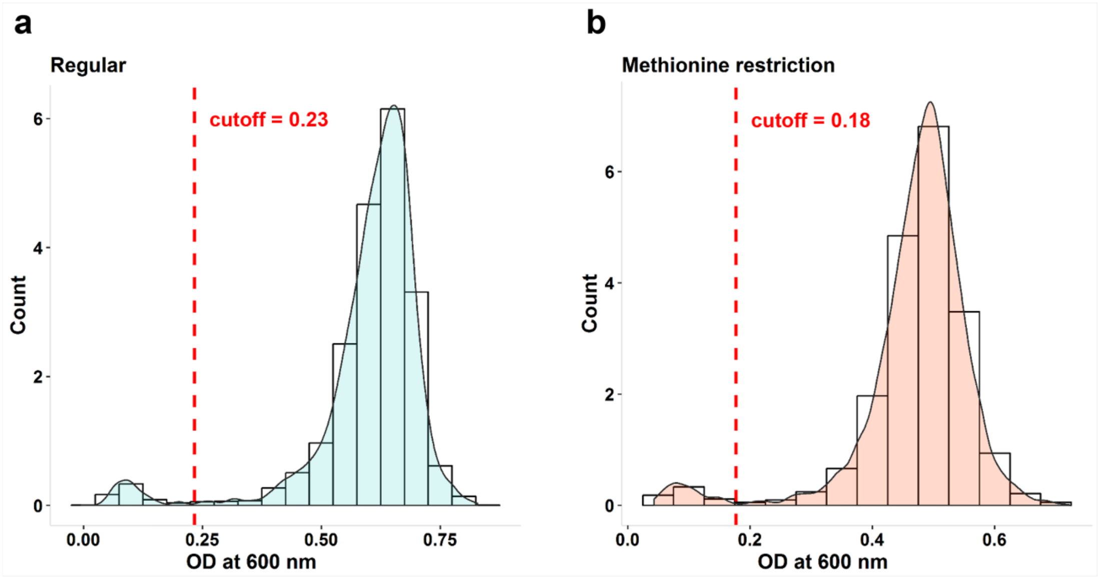

Figure S8. Histogram for OD_{600} of total screening yeast strains under regular (**a**) and MR (**b**). Cells with OD value under a third of wild-type in each condition (below red dotted line) are regarded as the growth-defective.
