# Supplementary material for: Sulfate assimilation regulates hydrogen sulfide production independent of lifespan and reactive oxygen species under methionine restriction condition in yeast
Source: Aging (Albany NY). 2019 Jun 29;11(12):4254–73. doi: 10.18632/aging.102050 (PMC6628990; doi:10.18632/aging.102050)
Supplement: Supplementary Tables [file aging-11-102050-s001.pdf]

Supplementary Tables

Table S1. Selected genes by comparison between wild-type and mutant (Analysis 1; 137 genes).

ORF name	Symbol	Fold-change (Mutant vs. Wild-type)	FDR-adj p-value (Mutant vs. Wild-type)
YLR027C	AAT2	-2.6	0.0300
YDR448W	ADA2	-4.9	0.0336
YOR128C	ADE2	-2.4	0.0496
YGR204W	ADE3	-2.0	0.0430
YGR061C	ADE6	-1.5	0.0306
YOL086C	ADH1	-6.7	0.0159
YMR083W	ADH3	-2.0	0.0251
YBR132C	AGP2	-4.5	0.0231
YGL105W	ARC1	-2.2	0.0430
YNL059C	ARP5	-4.5	0.0206
YOR141C	ARP8	-4.4	0.0175
YPR049C	ATG11	-2.2	0.0273
YDL113C	ATG20	-2.2	0.0251
YNL315C	ATP11	-2.9	0.0105
YOR026W	BUB3	-2.4	0.0248
YLR074C	BUD20	-4.4	0.0206
YCR047C	BUD23	-7.4	0.0228
YER014C-A	BUD25	-4.9	0.0170
YFL023W	BUD27	-2.5	0.0418
YGR262C	BUD32	-2.3	0.0357
YER061C	CEM1	-4.0	0.0175
YGL206C	CHC1	-2.7	0.0430
YPL241C	CIN2	-7.0	0.0083
YNL225C	CNM67	-2.1	0.0211
YPL172C	COX10	-2.3	0.0357
YMR256C	COX7	-3.9	0.0293
YJL172W	CPS1	-5.0	0.0210
YKL139W	CTK1	-2.4	0.0404
YJL006C	CTK2	-1.8	0.0228
YAL012W	CYS3	-11.6	0.0426
YKL054C	DEF1	-3.9	0.0206
YDL160C	DHH1	-2.9	0.0216
YCR034W	ELO2	-2.4	0.0306
YBR101C	FES1	-3.0	0.0211
YLR214W	FRE1	-2.0	0.0206
YAL035W	FUN12	-5.3	0.0175
YLR068W	FYV7	-2.5	0.0249
YOR205C	GEP3	-1.9	0.0176
YHR100C	GEP4	-1.5	0.0491
YER083C	GET2	-3.0	0.0032
YEL046C	GLY1	-3.8	0.0085
YGR102C	GTF1	-2.2	0.0494
YDR174W	HMO1	-3.1	0.0228
YER052C	HOM3	-4.7	0.0228

YCR020W-B	HTL1	-2.7	0.0206
YIL090W	ICE2	-3.4	0.0214
YEL044W	IES6	-3.5	0.0362
YMR035W	IMP2	-3.0	0.0231
YDR017C	KCS1	-1.6	0.0323
YFR001W	LOC1	-4.5	0.0083
YHR081W	LRP1	-2.2	0.0261
YJL124C	LSM1	-1.8	0.0367
YDR378C	LSM6	-2.2	0.0418
YKL143W	LTV1	-4.5	0.0175
YLR244C	MAP1	-2.3	0.0379
YOL076W	MDM20	-3.7	0.0211
YGL219C	MDM34	-1.8	0.0468
YKR069W	MET1	-2.4	0.0399
YFR030W	MET10	-3.4	0.0177
YGL125W	MET13	-10.1	0.0166
YIL128W	MET18	-2.7	0.0410
YNL277W	MET2	-6.7	0.0175
YOL064C	MET22	-11.9	0.0175
YJR010W	MET3	-2.8	0.0418
YJR137C	MET5	-2.7	0.0211
YER091C	MET6	-5.4	0.0149
YOR241W	MET7	-4.7	0.0175
YJR077C	MIR1	-1.8	0.0284
YNL076W	MKS1	-3.8	0.0231
YJR074W	MOG1	-2.7	0.0231
YOR201C	MRM1	-3.3	0.0280
YPL118W	MRP51	-2.2	0.0430
YBR122C	MRPL36	-5.9	0.0206
YPL173W	MRPL40	-2.0	0.0418
YPR100W	MRPL51	-1.9	0.0248
YIR021W	MRS1	-4.8	0.0166
YHR120W	MSH1	-3.7	0.0175
YDR432W	NPL3	-3.8	0.0206
YEL062W	NPR2	-2.7	0.0343
YHL023C	NPR3	-3.0	0.0379
YJR073C	OPI3	-3.3	0.0175
YER178W	PDA1	-1.7	0.0275
YOR036W	PEP12	-4.2	0.0121
YNR052C	POP2	-2.4	0.0228
YLR165C	PUS5	-2.7	0.0176
YDL104C	QRI7	-2.0	0.0287
YLR039C	RIC1	-1.8	0.0430
YCR028C-A	RIM1	-8.8	0.0206
YER070W	RNR1	-3.0	0.0382
YMR142C	RPL13B	-2.1	0.0305
YBR191W	RPL21A	-2.8	0.0482
YLR061W	RPL22A	-3.8	0.0125
YHR010W	RPL27A	-4.4	0.0228
YDL075W	RPL31A	-9.2	0.0206
YJL189W	RPL39	-4.5	0.0085

YLR448W	RPL6B	-1.8	0.0460
YDR382W	RPP2B	-2.1	0.0418
YLR048W	RPS0B	-1.9	0.0296
YHR203C	RPS4B	-1.6	0.0454
YOR096W	RPS7A	-3.0	0.0482
YBL025W	RRN10	-2.6	0.0206
YBR147W	RTC2	-2.4	0.0420
YOL067C	RTG1	-3.6	0.0175
YMR060C	SAM37	-2.0	0.0175
YLR403W	SFP1	-2.6	0.0468
YOR035C	SHE4	-2.0	0.0498
YGR112W	SHY1	-1.8	0.0369
YHL025W	SNF6	-3.2	0.0357
YJR104C	SOD1	-1.5	0.0348
YGL127C	SOH1	-1.6	0.0379
YOL052C	SPE2	-2.3	0.0357
YPR069C	SPE3	-2.8	0.0468
YOL148C	SPT20	-3.9	0.0231
YBR081C	SPT7	-2.7	0.0497
YHR041C	SRB2	-2.4	0.0261
YLR369W	SSQ1	-2.7	0.0302
YHR064C	SSZ1	-2.5	0.0228
YDR463W	STP1	-2.3	0.0356
YPL180W	TCO89	-2.0	0.0206
YOL072W	THP1	-4.3	0.0010
YHR167W	THP2	-2.3	0.0486
YPR133W-A	TOM5	-2.3	0.0357
YOR006C	TSR3	-5.7	0.0200
YBR173C	UMP1	-4.6	0.0228
YDL185W	VMA1	-1.8	0.0460
YBR127C	VMA2	-4.0	0.0345
YGR105W	VMA21	-2.3	0.0389
YEL027W	VMA3	-2.7	0.0410
YKL080W	VMA5	-3.4	0.0293
YGL095C	VPS45	-3.2	0.0306
YGR285C	ZUO1	-4.0	0.0287
YNL276C		-5.0	0.0175
YDR521W		-3.5	0.0121
YKL118W		-3.0	0.0265
YOR331C		-2.8	0.0166
YOR200W		-2.3	0.0357
YJR018W		-1.9	0.0273

Table S2. Selected genes by comparison between regular and MR condition (Analysis 2; 81 genes).

ORF name	Symbol	Ratio (MR/Regular)	FDR-adj p-value (MR vs. Regular)
YMR083W	ADH3	0.7	0.0259
YNL059C	ARP5	0.4	0.0043
YPR049C	ATG11	0.7	0.0164
YNL315C	ATP11	0.3	0.0047

YER014C-A	BUD25	0.5	0.0042
YFL023W	BUD27	0.5	0.0135
YLR226W	BUR2	0.6	0.0454
YOR125C	CAT5	0.6	0.0135
YGR062C	COX18	0.8	0.0125
YMR256C	COX7	0.6	0.0202
YJL172W	CPS1	0.2	0.0025
YDL160C	DHH1	0.4	0.0016
YDR069C	DOA4	0.7	0.0476
YGL240W	DOC1	0.7	0.0182
YCR034W	ELO2	0.7	0.0332
YBR101C	FES1	0.5	0.0040
YGR252W	GCN5	0.7	0.0227
YOR205C	GEP3	0.6	0.0152
YHR100C	GEP4	0.5	0.0026
YER040W	GLN3	0.7	0.0132
YOL049W	GSH2	0.5	0.0159
YGR102C	GTF1	0.5	0.0087
YEL059W	HHY1	0.8	0.0359
YER052C	HOM3	0.4	0.0043
YEL044W	IES6	0.4	0.0098
YHR081W	LRP1	0.6	0.0305
YJL124C	LSM1	0.7	0.0076
YOR221C	MCT1	0.7	0.0039
YGL219C	MDM34	0.6	0.0134
YKR069W	MET1	0.5	0.0159
YFR030W	MET10	0.3	0.0016
YGL125W	MET13	0.1	0.0014
YIL128W	MET18	0.4	0.0085
YNL277W	MET2	0.3	0.0039
YOL064C	MET22	0.1	0.0033
YJR010W	MET3	0.5	0.0089
YJR137C	MET5	0.5	0.0040
YER091C	MET6	0.2	0.0042
YOR241W	MET7	0.3	0.0032
YJR077C	MIR1	0.6	0.0290
YGL124C	MON1	0.6	0.0116
YOR201C	MRM1	0.5	0.0247
YKR085C	MRPL20	0.8	0.0333
YPR100W	MRPL51	0.7	0.0314
YDL107W	MSS2	0.9	0.0427
YEL062W	NPR2	0.6	0.0094
YHL023C	NPR3	0.6	0.0372
YER178W	PDA1	0.6	0.0060
YNR052C	POP2	0.7	0.0183
YDL006W	PTC1	0.7	0.0262
YER070W	RNR1	0.5	0.0163
YFL036W	RPO41	0.5	0.0078
YHR203C	RPS4B	0.8	0.0178
YDR502C	SAM2	0.8	0.0135
YCL010C	SGF29	0.5	0.0188

YGR112W	SHY1	0.7	0.0394
YDL047W	SIT4	0.6	0.0127
YCR024C	SLM5	0.9	0.0345
YLR025W	SNF7	0.8	0.0431
YJR104C	SOD1	0.9	0.0332
YOL052C	SPE2	0.6	0.0233
YJL127C	SPT10	0.6	0.0229
YHR178W	STB5	0.6	0.0129
YDR463W	STP1	0.4	0.0183
YCL008C	STP22	0.6	0.0095
YHR167W	THP2	0.5	0.0062
YPR133W-A	TOM5	0.6	0.0142
YOR187W	TUF1	0.7	0.0091
YHR111W	UBA4	0.7	0.0324
YDR470C	UGO1	0.5	0.0100
YBR127C	VMA2	0.6	0.0332
YGR105W	VMA21	0.6	0.0083
YEL027W	VMA3	0.4	0.0070
YLR447C	VMA6	0.6	0.0266
YEL051W	VMA8	0.5	0.0159
YDR080W	VPS41	0.6	0.0293
YML009W-B		0.5	0.0017
YNL276C		0.5	0.0062
YJR018W		0.6	0.0196
YKL118W		0.6	0.0454
YGR219W		0.9	0.0278

Table S3. Median CLS of wild type and deletion mutants on the regular and MR conditions.

Strain	Median CLS on the regular (days)	Median CLS on the MR (days)	p-value (Regular vs. MR)
Wild type	10.1 ± 0.04	13.0 ± 0.95	0.0896
met1Δ	4.9 ± 0.02	12.2 ± 1.37	0.0336
met3Δ	7.2 ± 0.06	23.4 ± 0.18	0.0028
met5Δ	3.2 ± 0.58	20.3 ± 0.78	0.0001
met10Δ	4.9 ± 0.02	13.6 ± 0.84	0.0611

* Data indicate mean ± SEM. P-value was calculated by two-tailed Student's *t*-test.

Table S4. Composition of synthetic complete (SC) media.

**Component**	**Amount per liter (L)**
Yeast nitrogen base without amino acid	6.7 g
Dextrose	20 g
L-adenine	10 mg
L-arginine	50 mg
L-aspartic acid	80 mg
L-histidine	20 mg
L-isoleucine	50 mg
L-leucine	100 mg

L-lysine	50 mg
L-methionine	50 mg (for regular) or 5 mg (for MR)
L-phenylalanine	50 mg
L-threonine	100 mg
L-tryptophan	50 mg
L-tyrosine	50 mg
L-uracil	20 mg
L-valine	140 mg
